# A genetically *hmgb2* attenuated blood stage *P. berghei* induces crossed-long live protection

**DOI:** 10.1371/journal.pone.0232183

**Published:** 2020-05-07

**Authors:** Sylvie Briquet, Nadou Lawson-Hogban, Roger Peronet, Salaheddine Mécheri, Catherine Vaquero

**Affiliations:** 1 Sorbonne Université, Centre d’Immunologie et des Maladies Infectieuses (CIMI-Paris), Paris, France; 2 INSERM, U1135, CIMI-Paris, Paris, France; 3 CNRS, ERL 8255, CIMI-Paris, Paris, France; 4 Unité de Biologie et Génétique du Paludisme, Institut Pasteur, Paris, France; 5 Centre National de Recherche Scientifique ou CNRS, Unité de Recherche Associée 2581, Paris, France; Instituto Rene Rachou, BRAZIL

## Abstract

Due to the lack of efficiency to control malaria elicited by sub-unit vaccine preparations, vaccination with live-attenuated *Plasmodium* parasite as reported 70 years ago with irradiated sporozoites regained recently a significant interest. The complex life cycle of the parasite and the different stages of development between mammal host and anopheles do not help to propose an easy vaccine strategy. In order to achieve a complete long-lasting protection against *Plasmodium* infection and disease, we considered a genetically attenuated blood stage parasite in the *hmgb2 gene* coding for the high-mobility-group-box 2 (HMGB2). This *Plasmodium* protein belongs to the HMGB family and hold as the mammal proteins, a double life since it acts first as a nuclear factor involved in chromatin remodelling and transcription regulation and second, when secreted as an active pro-inflammatory alarmin protein. Even though the number of reports on whole living attenuated blood stage parasites is limited when compared to attenuated sporozoites, the results reported with *Plasmodium* KO parasites are very encouraging. In this report, we present a novel strategy based on pre-immunization with *Δhmgb2Pb*NK65 parasitized red blood cells that confer long-lasting protection in a murine experimental cerebral malaria model against two highly pathogenic homologous and heterologous parasites.

## Introduction

Even though WHO claims (World malaria report, 2018) that malaria threat was decreasing there is still around 500.000–700000 deaths per year of children and pregnant women dying of severe malaria (hyperparasitaemia and anaemia as well as cerebral malaria). *Plasmodium falciparum* remains a noteworthy killer essentially in sub-Saharan Africa and probably currently amplified by politic instability. In addition, the disease is spreading worldwide owing to traveller mobility and to increased resistance towards both parasites and drugs. On the other hand, the fight against malaria is hampered by the lack of effective vaccines capable to control the development of the parasites and the onset of disease. The first attempts to develop a vaccine date back 70 years based on whole radio-inactivated sporozoites [1]. Thereafter, the development of molecular biology, *Plasmodium* genomic, DNA sequencing and protein investigations [2] shifted the *Plasmodium* community interest to sub-unit protein vaccine [3]. However, since these vaccine preparations did not prove a proper protection [4], the attenuated living parasites regain a noteworthy interest [5, 4]. The main studies were developed for attenuated sporozoites with still discouraging results.

As for the living red blood cell (RBC) vaccines, the first report [6] showed that a low amount as low as 30–300 *P*. *falciparum* parasitized RBC (iRBC) infected with the 3D7 *Plasmodium* live parasites when injected four times to five volunteers followed by an anti-malarial treatment triggered a some protection against an homologous challenge. Thereafter, several trials were achieved with living parasites followed by antimalarial treatment [7, 8] as well as with chemically attenuated *P*. *falciparum* [7]. In addition, several genetically attenuated parasites with defects in replication capacity have been shown to lead to self-resolving infections leading possibly to potent and long-lasting protection in various murine models against both erythrocytic [9, 10, 11] and pre-erythrocytic [4, 12, 13, 14] challenges. However, these studies provided only limited information regarding the immunological mechanisms that confer protection except a recent study which provided a detailed characterization of the immune mechanisms underlying the long-lasting protective immunity provided by the HRF-deficient *Pb*NK65 parasites [15]. These immunological changes produced by living parasites that might potentially trigger long lasting protection was also studied in human after *P*. *falciparum* infection [16, 7].

The HMGB proteins members of High Mobility Group family are known in high eukaryotes to encompass a double life, when in the nucleus acting as remodelling chromatin and transcription factor, and when actively secreted acting as pro-inflammatory factors involved in lupus, septic shock, rheumatoid arthritis, cancer, etc. We reported that two *Plasmodium* HMGB behave as chromatin remodelling proteins [17] and as a late pro-inflammatory factor leading to experimental cerebral malaria (ECM) onset in a murine model. Actually, in contrast to WT *PbANKA* parasite that triggers mouse death within 7–8 days, mice infected with *hmgb2*-deleted parasite (*Δhmgb2 Pb*ANKA) showed an increased survival concomitantly (60%) with a decrease of parasite sequestration and haemorrhagic foci in the brain [18, 19]. In addition, the antibodies raised against the HMGB protein triggered protection pathogenic parasite infection [20]. We assumed that HMGB might be a promising vaccine candidate which may act if compromised at the level of i) gene expression preventing the development of the parasite, ii) host immune response preventing the host inflammatory response and therefore the disease. Herein, we provide evidence that a long-lasting sterile protection is triggered *via* a genetically attenuated parasite (GAP), *Δhmgb2 Pb*NK65, against infection with highly pathogenic homologous and heterologous parasites inducing severe anaemia and cerebral malaria, respectively.

## Materials and methods

### Ethics statement

Depending on the experiments death of mice was considered as endpoints for 1. C57/Bl6 infected with the two pathogenic parasites *Pb*ANKA and *Pb*NK65 dying from cerebral malaria or hyperparasitaemia and 2. mice eliciting long term protection euthanasia was performed by lethal sodium pentobarbital anaesthesia and all efforts were made to minimize suffering. All animal care and experiments involving mice described in the present study were approved by the Direction Départementale des Services Vétérinaires de Paris, France (permit A75-13-01), and performed in compliance with institutional guidelines and European regulations, (http://ec.europa.eu/environment/chemicals/lab_animals/home_en.htm).

### Mice

C57BL/6 female 6–8 weeks old mice were purchased from Janvier and Charles River Laboratories, respectively.

### Gene

ID of the murine *hmgb2* is PBANKA_071290.

### Parasites

Two highly pathogen parasites were used: *P*. *berghei* ANKA (*Pb*ANKA MRA-867) that kills C57Bl/6 within 7–10 days of experimental cerebral malaria (ECM) and *P*. *berghei* NK65 (*Pb*NK65 MRA-268) that kills C57Bl/6 within 25–30 days of hyperparasitaemia and anaemia. The disruption of the *hmgb2* gene in *Pb*NK65 background was performed with 5’ and 3’ gene targeting KO vector designed and produced as detailed for *Pb*ANKA [18]. Wild type *Pb*NK65 blood stages parasites were transfected using standard transfection methods [21]. Positive selection for successful integration of the targeting plasmid was carried out by pyrimethamine administration. Clonal *hmgb2 deficient* parasite populations from *Pb*NK65 strain were obtained by limiting dilution into 20 naive Swiss mice and confirmed by genotyping PCR and southern blotting. Mice were inoculated with murine red blood cells (RBCs) infected with *Pb*NK65 and its *hmgb2*-deficient counterpart *Δhmgb2 Pb*NK65 and occasionally with *Pb*ANKA as a highly pathogenic heterologous parasite control.

### Murine experiments

In all experiments, RBCs infected with *P*. *berghei* NK65 or its knockout counterpart *Δhmgb2 Pb*NK65 were used to infect 5 to 10-week-old C57BL/6 mice as previously reported [18].

For the priming process, the C57BL/6 mice were infected by intraperitoneal (i.p.) inoculation of 10^5^ *Δhmgb2 Pb*NK65 iRBCs. In one experiment the parasite concentration was lowered up to 10^3^ iRBC. Parasitaemia of *P*. *berghei* NK65 and derived KO parasites was determined by growth of WT by microscopic examination of Giemsa-stained thin blood smears. Parasitaemia was measured by counting 3,000 red blood cells and expressed as the percentage of total parasitized erythrocytes.

For the challenging-protection experiments the same mice were infected at different times post priming with 10^5^ iRBC infected with the two WT highly pathogenic parasites *Pb*NK65 and *Pb*ANKA. Experimental cerebral malaria, hyperparasitaemia and survival of the primed mice were examined all through the experiments (see Fig 2).

**Fig 1:**
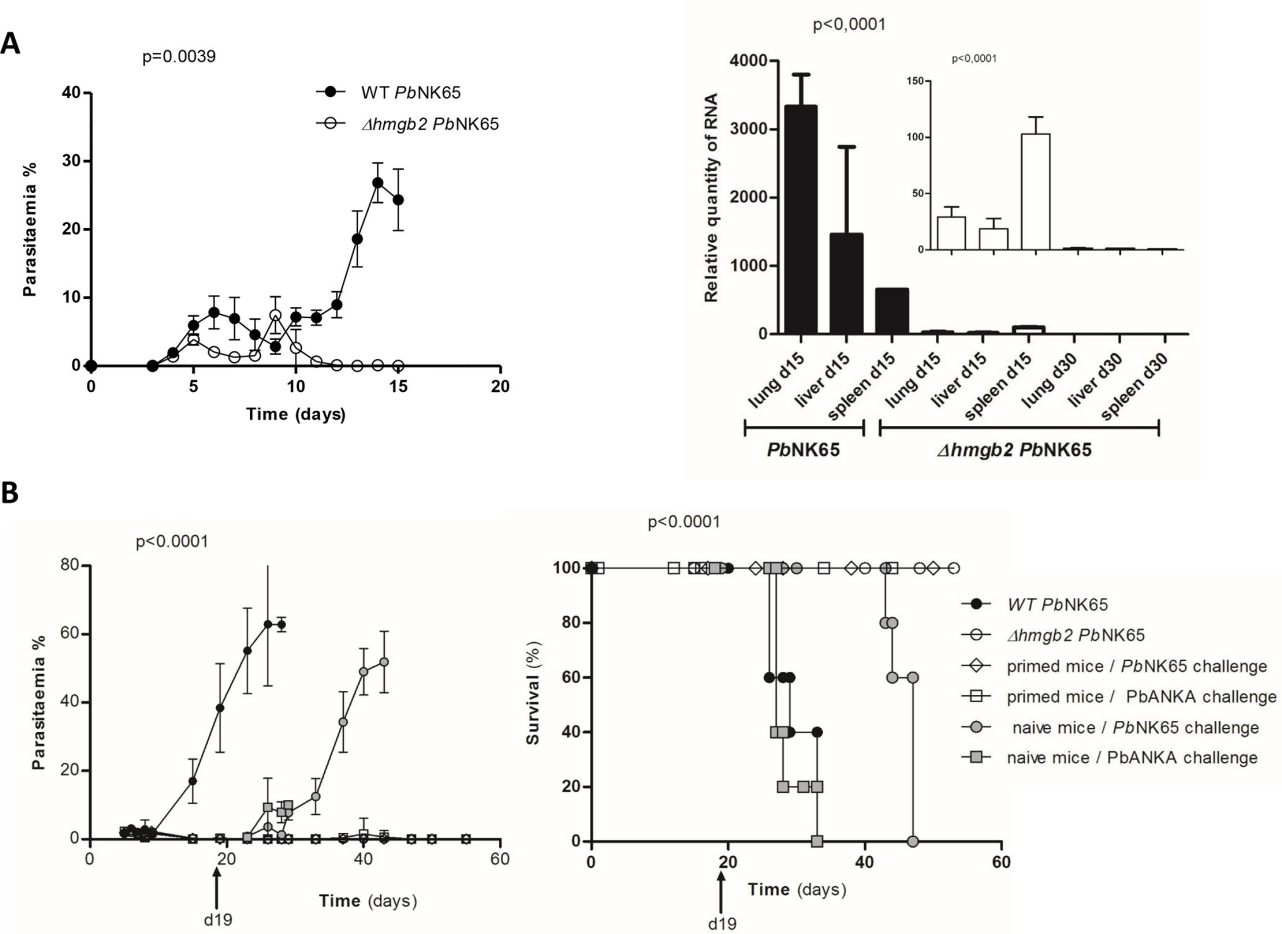
Development of *Δhmgb2 Pb*NK65 parasite in C57Bl/6 mice (A) and survival of *Δhmgb2 Pb*NK65 primed mice (B). **A.** Left: mice were infected by 10^5^ WT *Pb*NK65 and *Δhmgb2 Pb*NK65 RBC. Parasitaemia was monitored by microscopic counting every days up to d15 post infection. Right: analysis of parasite 18S RNA by RTqPCR in diverse organs (lung, liver, spleen) of the aforementioned mice at d15 or d30 post-infection. The insert corresponds to the zoom of the results obtained at d15 and d30 post infection. **B.** Survival of the mice primed with 10^5^ *Δhmgb2 Pb*NK65 iRBC was monitored after a challenge performed at d19 with both (10^5^) WT *Pb*NK65 (n = 5) and *Pb*ANKA (n = 5) parasites. Survival of naive mice (n = 5) infected with both lethal parasites was also analysed. The symbols are presented right part of the figure.

**Fig 2:**
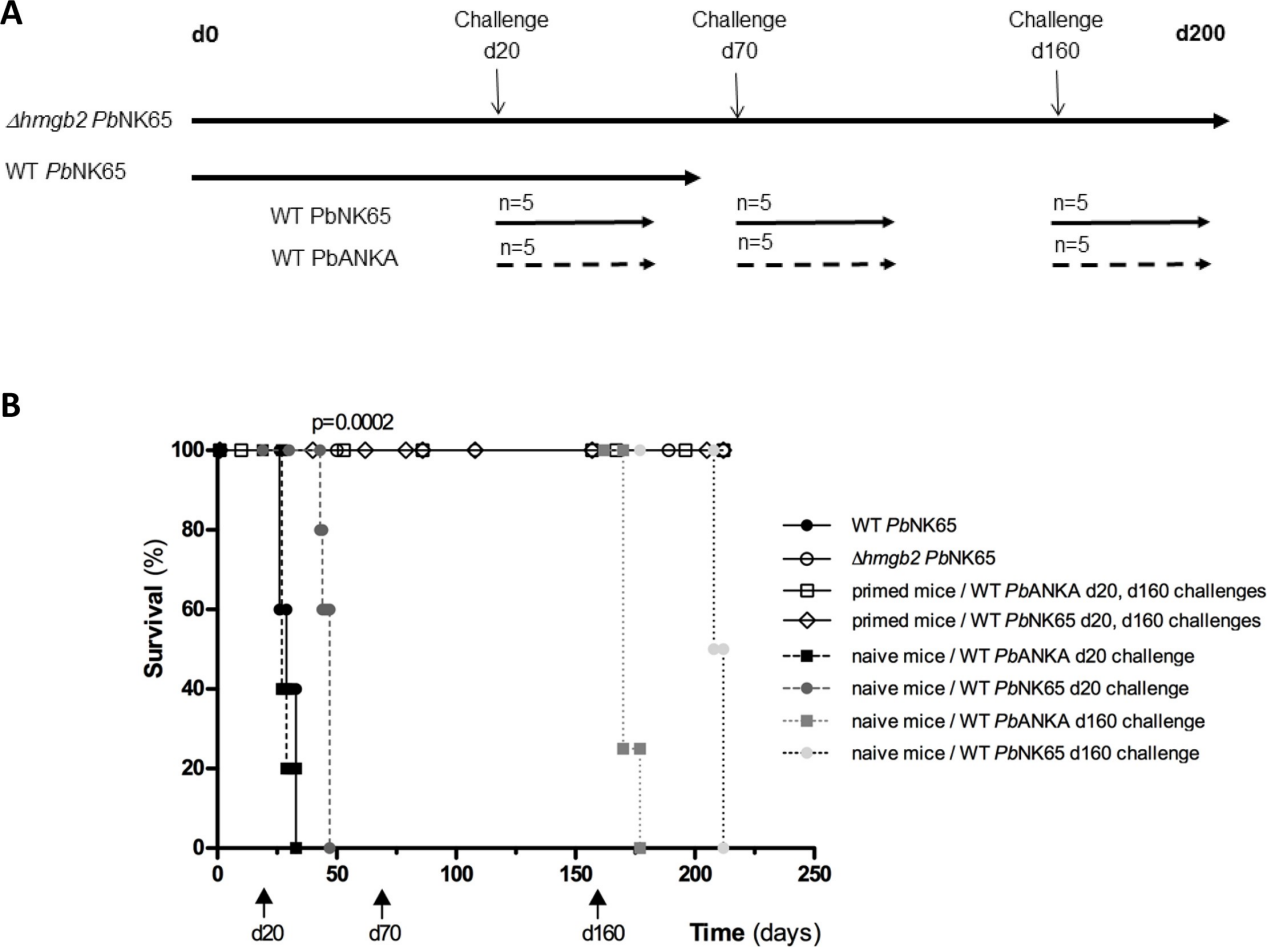
Long sterile protection triggered by *Δhmgb2 Pb*NK65 priming against both pathogenic parasites. **A.** Cartoon of the experimental process. Mice (6 weeks old) were either primed with 10^5^ *Δhmgb2 Pb*NK65 iRBC or not. At d20, d70 and d160 post-priming the mice were infected with either WT *Pb*NK65 or WT *Pb*ANKA. **B.** Survival of primed *hmgb2 Pb*NK65 mice (n = 5) challenged subsequently at d20, d70 (not presented for simplification) and d160 (arrow) post–priming with the two highly pathogenic parasites WT *Pb*NK65 or WT *Pb*NK65 was monitored. Also, as previously stated survival of naive mice (n = 5 was analysed at d20, d70 (not presented) and d160 after infection with WT *Pb*NK65 and WT *Pb*ANKA as a control of the pathogenicity of both WT parasites. The symbols are presented in the right part of the figure.

All protected mice were euthanized after 50 (Fig 1) or 200 days (Fig 2) with lethal sodium pentobarbital anaesthesia in contrast to control mice dying from of hyperparasitaemia or cerebral malaria. In addition, the control mice infected with both WT parasites *Pb*NK65 and *Pb*ANKA were monitored for hyperparasitaemia and for clinical symptoms of cerebral malaria, including hemi-paraplegia, deviation of the head, tendency to roll over on stimulation, ataxia and convulsions; one of these specific criteria allowed us to determine whether euthanasia was required for a given mouse.

### Preparation of total RNA and reverse transcription-quantitative PCR (RT-qPCR) transcript analysis

At different times post-priming (d15 and d30), total RNAs were extracted from lungs, liver and spleens removed from C57BL/6 infected mice. RNA preparation and primer sequences have been detailed in [18]. Each RNA preparation was analysed by real-time RT-qPCR in an MX 3005P cycler (Stratagene) for the 18S parasite RNA, using SYBR green Jumpstart*Taq*ReadyMix (Sigma). Expression was normalized with the hypoxanthyl-guanine phosphoribosyltransferase (*hprt*) transcript.

### Statistical analysis

Differences in mouse survival were evaluated by the generation of Kaplan-Meier survival plots and log rank analysis. Statistical significance and parasite loads were assessed by the Student *t* test or non-parametric analysis using Kruskal-Wallis to compare means between the 3 to 8 groups of growth rates, survival curves and transcript expression levels. Dunn’s post-test was applied to analyse the effect of *pbhmgb2* deletion. All statistical tests were performed with GraphPad Prism 7. *P* values <0.05 were considered statistically significant for each test. In vivo experiments were performed at list 3 times, with 5 to 10 mice per experiment.

### Nucleotide sequence accession numbers

The sequences of the *P*. *berghei* high-mobility-group protein gene *pbhmgb2* (PBANKA_071290) and the *Pb*ANKA rRNA-encoding gene berg06_18s are available in PlasmoDB (http://plasmodb.org/plasmo/).

## Results

### *Δhmgb2 Pb*NK65 does not develop properly in C57Bl/6 mice and generates cross protection

The C57Bl/6 mice were infected with 10^5^ red blood cells of either *Pb*NK65 or its *hmgb2* disrupted *Δhmgb2 Pb*NK65 counterpart. The disruption procedure of the *hmgb2* gene was already described in a previous report [18]. The parasitaemia was monitored by microscopic counting of Giemsa coloured blood smears every other day for 3 clonal populations compared to the WT parasite. One representative clone was selected for this study. After an initial development, clearance of the *Δhmgb2 PbNK65* parasite was observed at d15 post-infection (i.p.) in contrast to the WT parasite which completed its development until mouse death from hyperparasitaemia (Fig 1A, left panel) (*p* = 0.0039). In addition, even though the parasite was no more detected in the circulating blood, we verified that the mouse organs were completely cleared of iRBC. We considered the parasite RNA (biomass) in lungs, liver and spleen of infected mice. As shown in Fig 1A (right panel) the *Δhmgb2 Pb*NK65 iRBC was hardly detectable at d15 post-infection in any of the organs as measured by RT-qPCR of parasite 18S RNA in contrast to the mice infected with the WT *Pb*NK65. In addition, at d30 the KO parasite was no more detected. The absence of iRBC in the peripheral blood is consistent with the absence of the parasite in the organs and precludes the possibility of iRBC sequestration.

We then looked if priming with one shot of disrupted parasites was able to interfere, in addition to the homologous WT parasite with the heterologous WT pathogenic *Pb*ANKA parasite. Mice were first infected as previously stated with 10^5^ *Δhmgb2 Pb*NK65 and after clearance of the KO parasites from the blood stream, one challenge was performed at d19 post-priming with 10^5^ red blood cells infected with either WT *Pb*NK65 or WT *Pb*ANKA. We looked at the parasite growth (Fig 1B left panel) and mouse survival (Fig 1B right panel) of the naive and primed mice. In these primed mice (d19) both WT parasites do not develop up to d55 post infection in contrast to naive mice dying either within 28 days or 9–14 days of hyperparasitaemia or cerebral malaria, respectively. This control was performed to assess the pathogenicity of both WT parasite preparations. Parasitaemia was monitored by microscopic counting as aforementioned. Interestingly, survival of all primed mice infected after clearance with 10^5^ iRBC with the two highly pathogenic wild type *Pb*NK65 and *Pb*ANKA (Fig 1B right panel) was observed up to d55 when the mice were sacrificed, in contrast to the death of all naive mice. In summary, all primed mice were protected from subsequent infection with the two highly pathogenic parasites.

### One single delivery of *Δhmgb2 Pb*NK65 iRBC was able to confer a long-lasting immunity

It was questioned whether one single priming with life *Δhmgb2 Pb*NK65 *parasites* (10^5^ iRBC i.p. as previously stated) was able to induce a long-lasting protective immunity against multiple subsequent challenges with the two lethal WT parasites. The schematic protocol is presented in Fig 2A indicating the three challenges carried out at d20 as aforementioned, d70 and d160 with the same amount of iRBC in primed and naive mice. The one shot *Δhmgb2 Pb*NK65-primed mice remained completely parasite-free and all mice survived after the three successive challenges with the two highly lethal homologous and heterologous parasites (Fig 2B). All mice were still alive up to 7 months (210 days) a time at which the mice were sacrificed. For simplification, the curves assessing the mouse survival after the second challenge carried out at d70 as d20 and d160 challenges were not presented in the figure. At each time challenge, control naive mice were infected with both WT parasites, and they consistently died around day 25–30 or day 7–13 following infection with WT *Pb*NK65 or *Pb*ANKA parasites, respectively, assessing the pathogenicity of both parasite preparations and suggesting that the mice age does not account for the effect observed.

### Lower amounts of *Δhmgb2 Pb*NK65 iRBC were capable to confer sterile protection

**Fig 3:**
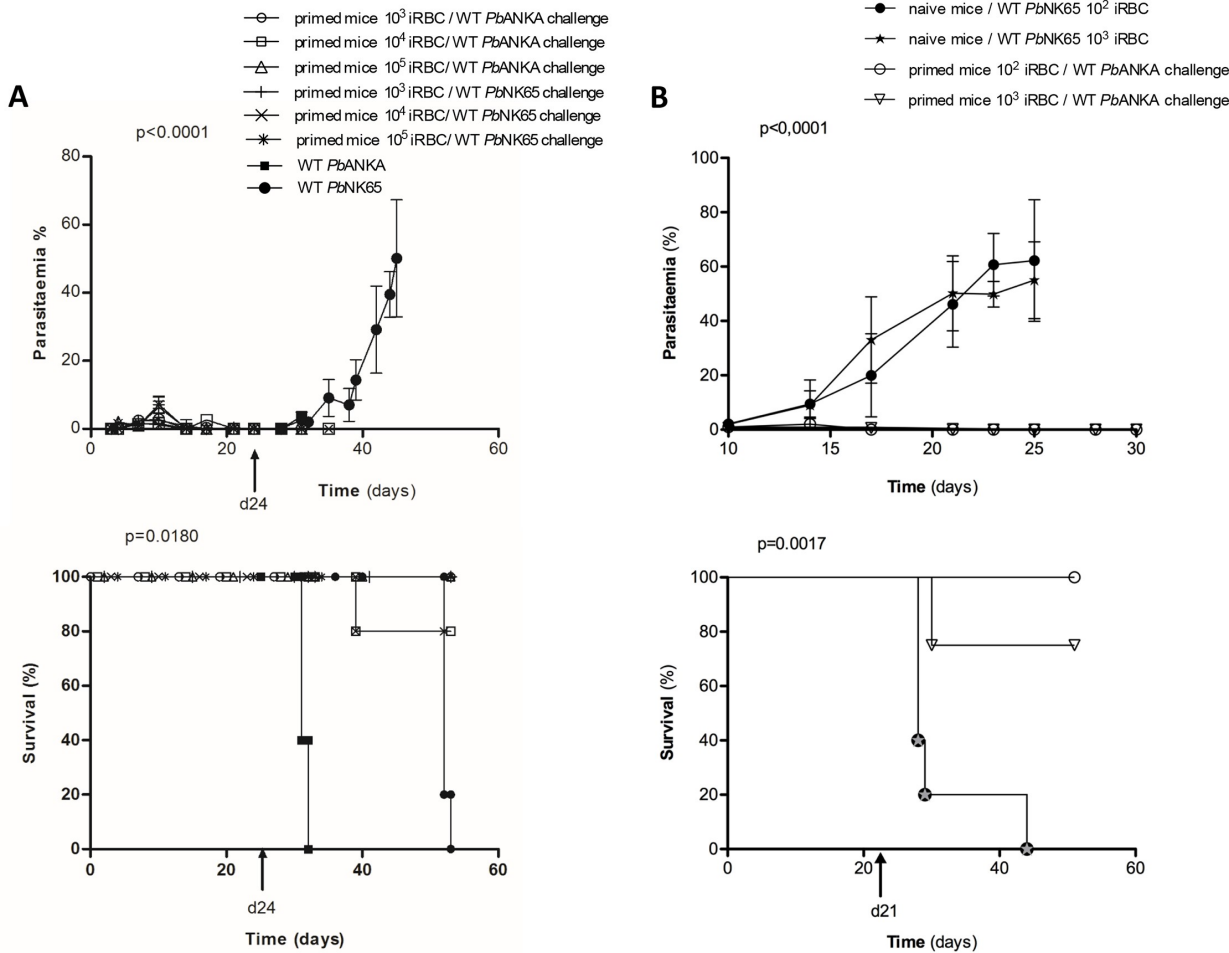
Dose response experiment to evaluate the number of *Δhmgb2 Pb*NK65 RBC capable to trigger protection against the lethal wild type parasites. Three sets of mice (n = 5) were primed with different concentration of *Δhmgb2 Pb*NK65: iRBC (10^5^, 10^4^ and 10^3^). At d24 post priming the three sets of mice were subsequently challenged with the two highly pathogenic parasites either 10^5^ WT *Pb*NK65 or WT *Pb*ANKA. **A.** Parasitaemia was monitored as in Fig 1. **B.** Survival was monitored as depicted in previous figures. The symbols are summarized in the right part of the figure.

It is essential to evaluate *via* a single dose response experiment the lowest number of *Δhmgb2 Pb*NK65 iRBC necessary to trigger a long-lasting protection that in turn impair the development of the challenging lethal parasites. First, three different amounts of *Δhmgb2 Pb*NK65 iRBC parasite were used from 10^5^ as aforementioned and 10^4^ and 10^3^ for priming infection. These three sets of primed mice were thereafter challenged at d24 with 10^5^ iRBC infected with the WT *Pb*NK65 and *Pb*ANKA parasites. Parasitaemia in Fig 3A (top panel) reveals the lack of growth observed with the different concentrations used for priming and challenged thereafter at d24 with the two WT *Pb*NK65 and WT *Pb*ANKA. In contrast to what happens in naive mice. In addition, all mice primed with 10^5^ and 10^3^ KO iRBC survived up to 53 days (Fig 3A, bottom panel) when only 80% of the mice primed with 10^4^ iRBC *Pb*ANKA survived. In contrast, naive mice infected at d24 with the WT *Pb*NK65 and *Pb*ANKA died within 29 and 7–8 days post-infection of hyperparasitaemia and cerebral malaria, respectively assessing the pathogenicity of the WT parasites. Second, two different doses 10^2^ and 10^3^ of GAP were used and do not develop in naive mice in contrast to the same amounts of WT *Pb*NK65 dying at d25 post-infection (Fig 3B, top panel). Actually, these two sets of primed mice survived up to 51 days when they were sacrificed when challenged at d21 with10^5^ WT *Pb*ANKA even though a break-through was observed with 10^3^ GAP priming. All mice primed with 10^2^ GAP survived (Fig 3B, bottom panel) highlighting that a very low amount one hundred of GAP was capable of triggering long lasting protection. Again, the pathogenicity of the WT parasite was verified and killed the naive mice in around 23 days.

## Discussion

The difficulty to obtain an efficient vaccine to combat malaria resides in the complex life of the *Plasmodium* parasite and its antigenic variation. Different stages of its life cycle in humans either at the level of asymptomatic pre-erythrocytic or erythrocytic development responsible for the disease can be addressed. Since the sub-unit vaccine did not lead to efficient vaccine preparations, whole attenuated living parasites regained interest and were tested for their capacity to impair cell invasion or active parasite development mainly at the pre-erythrocytic stage thoroughly evaluated in murine models. It is of note that the first report for attenuated life sporozoites dates back to 1967 [1]. Many reports dealing with GAP sporozoites were published in different murine models (see Introduction) and shown to cause self-resolving infections leading to protection. However, in the context of vaccination in humans the genetically attenuated sporozoites are difficult to produce and huge amounts have to be injected to human several times (IV) impairing a large-scale vaccination [22]. Vaccination with whole red blood cells infected with chemically attenuated parasites was also investigated in murine models and some cross-protection was occasionally observed [16, 23].

Our knowledge of the function of HMGB in humans and in *Plasmodium* led us to envisage that the parasite proteins might play a major role in the parasite cycle and malaria since when in the nucleus it acts as a remodelling factor involved in gene regulation [17] and since when released out of the parasite it acts as an efficient immune activator implicated in the disease [24, 18]. That is why we assumed that the *Plasmodium* HMGB proteins might be an effective target for vaccination and that the genetically inactivated gene parasite might trigger protection. Actually, Tsuboi’ team reported that, among the 1827 recombinant proteins produced in the wheat germ lysate and evaluated with 51 plasma samples from adults leaving in Malian low malaria transmission area, the *P*. *falciparum* HMGB1 and HMGB2 proteins encompassed a marked immune reactivity with a potential protective efficacy elicited by the corresponding antibodies. Within all the proteins assayed, the PfHMGB2 protein is among the strongest scores [25]. These results are in good agreement with our results showing that in mice the antibodies raised against the HMGB proteins were capable to control parasite infection [20].

We observed that *Δhmgb2 Pb*NK65 were capable to control subsequent parasite infection. In the mice, the *Δhmgb2 Pb*KK65 was cleared from the blood stream within 20 days with no residual RNA parasite in the organs tested (Fig 1A). However, the low development of the parasite was capable to induce an immune response capable to subsequently control the parasite development of homologous and heterologous lethal parasites used as challenge (Fig 1B). This protection was long-lasting since three successive challenges at 20, 70, and 160 days post-priming did not induce the death of mice (Fig 2). This long-lasting protection was observed in mice after one shot of 10^5^ *Δhmgb2 Pb*KK65 iRBC parasite preparations subsequently challenged with 10^5^ iRBC of the highly lethal *PbNK65 and Pb*ANKA parasites. The pathogenicity of both WT parasites was controlled at every challenge. Finally, one shot of low amount of iRBC (1000) was able to protect also the mice against two subsequent challenges (Fig 3A) and even a lower amount 100 *Δhmgb2 Pb*KK65 was capable to prime effectively the mice that survive *Pb*ANKA infection (Fig 3B). All these data pave the way of an innovative vaccine strategy taking into account the novelty of the HMGB proteins.

Several reports of Good’ team showed that cellular immune responses and protection were induced by different chemically attenuated parasites in various murine models [16, 23] and in humans [6, 26]. Actually, a long-lasting cellular immune response was observed in a small number of human volunteers induced by chemically attenuated *P*. *falciparum* followed by a drug treatment [7]. However, no parasite challenge was analysed thereafter and need further work to establish if this reactive immune response will be cross protective in humans. That is why our report on long-lasting cross protection determined by murine infection of a genetically attenuated parasite lacking HMGB2 protein might be the first step to propose a novel strategy for malaria vaccination leading to a clinical study of *hmgb2* inactivated *P*. *falciparum*). It is of note that the dual function of this protein is an important and stimulating challenge for a new strategy to control malaria. This gene inactivation by specific antibodies and/or small inhibitor molecules is of importance since the gene product is be-functional for a better control of i. parasite development and its transmission as well as ii. immunological responses responsible for malaria.
